# Prospective Mental Images: A Transdiagnostic Approach to Negative Affectivity and Mood Dysregulation among Borderline Personality Disorder and Depression

**DOI:** 10.3390/bs14020081

**Published:** 2024-01-23

**Authors:** Julia Kroener, Caroline Schaitz, Zrinka Sosic-Vasic

**Affiliations:** 1 Department of Applied Psychotherapy and Psychiatry, Christophsbad Goeppingen, Jahnstraße 30, 73035 Goeppingen, Germany; 2 Department of Psychiatry and Psychotherapy III, University Clinic of Ulm, Leimgrubenweg 12–14, 89075 Ulm, Germany; 3 Psychotherapeutic Outpatient Facility, Medical School Berlin, Rüdesheimer Straße 50, 14197 Berlin, Germany

**Keywords:** borderline personality disorder, depression, negative affectivity, non-suicidal self-injury, NSSI, prospective intrusive imagery, transdiagnostic

## Abstract

There is initial evidence that patients diagnosed with Borderline Personality Disorder (BPD) experience intrusive prospective mental images about non-suicidal self-injury (NSSI). These images, in turn, are associated with the conduct of NSSI. As the negative emotional valence of intrusive images has been established across clinical disorders, negative affectivity might play a key role linking mental imagery and psychopathology. Therefore, the present study aimed to investigate the possible mediating role of symptoms of depression as a proxy for negative affectivity linking intrusive prospective imagery to psychopathology in patients diagnosed with BPD. A total of 233 participants (84 diagnosed with MDD, 66 diagnosed with BPD, 83 healthy controls) completed questionnaires on negative affectivity (BDI-II) and prospective intrusive imagery (IFES-S). Before controlling for negative affectivity, there was a positive correlation between group and intrusive prospective imagery, indicating that healthy participants displayed lower amounts of intrusive prospective images in comparison to patients diagnosed with MDD or BPD. After entering negative affectivity as a mediator, the variable group was no longer associated with intrusive prospective images; however, negative affectivity showed a strong and positive relationship with the group on one side, and intrusive prospective imagery on the other, indicating that negative affectivity mediates the association between intrusive prospective images and clinical disorders. The presented findings point towards a mediating role of negative affectivity in the manifestation of intrusive prospective imagery, not only within BPD, but also in patients with MDD. The possibility of intrusive images acting as a transdiagnostic feature, where negative affectivity and mood dysregulation are at the core of the clinical disorder, are being discussed.

## 1. Introduction

The ability to use mental images is a fundamental, daily human ability. Memories, particularly autobiographical memories, are triggered more spontaneously via mental imagery than through verbal–linguistic representations [1,2,3]. Remembering our first kiss, for example, engages numerous sensory modalities: we see, hear, smell, and taste the (ideally favorable) situation at the time as “seeing in the mind’s eye” or “hearing in the mind’s ear” [4]. In line with this idea, mental images are characterized as perceptual experiences that occur in the absence of actual external stimuli. They manifest in a multi-sensory format remarkably similar to real-life experiences [4,5]. Mental images differ from mere cognitions. These cognitions are represented in a rather verbal–linguistic fashion and lack multi-sensory information, resulting in less affective engagement than mental images [6,7]. 

Because mental images influence emotional, motivational, and behavioral processes, they are thought to play an important role in the maintenance and exacerbation of psychopathology (for review, see [8]). Mental images might be thought of as “amplifiers” on an emotional level [6,9,10,11]. For example, studies experimentally manipulating verbal vs. imagery representations revealed that mental imagination led to stronger affective responses than verbal–semantic representations of the same material [3]. At the motivational level, results show that when stimuli are presented as mental images, their attractiveness and desirability increases [12]. At the behavioral level, mental images have been identified to direct behavior. Specifically, they increase the probability of future behavior once this behavior has been imagined [13]. 

Over the last decade, evidence has accumulated pointing to the clinical importance of mental images in a wide range of mental disorders, including affective disorders and Borderline Personality Disorder (BPD; [8,14,15]). Several studies have shown that while patients suffering from depression generate fewer pleasant mental images [7,16,17], they also produce more intrusive, aversive mental images [18]. In patients suffering from depression, both retrospective aversive images, such as memories of negative experiences [19], and prospective mental images [20], such as imaginations about one’s own (future) suicide, have been observed. Recent studies have successfully applied treatment approaches based on this knowledge, such as imagery trainings consisting of positive, prospective self-instructions [21,22,23,24,25]. Furthermore, recent findings suggest that utilizing mental imagery could be an effective method to reduce symptoms of depression [26].

In addition to these findings on mental imagery in depression, recent studies have demonstrated that self-harming behaviors are related to the occurrence of mental images in BPD and self-harming youths [15,27]. Higher rates of non-suicidal self-injury (NSSI) were associated with greater rates of distressing negative images in a web-based survey of clinical and non-clinical individuals [28]. In addition, the urge to self-harm was positively associated with thinking in images [29]. These images precede, and, therefore, announce, the subsequent self-harm [30]. A pilot study of ten self-injuring adults revealed that the first mental image of self-harm occurred directly after the first attempt to self-harm, increasing the urge to self-injure [31]. There is preliminary evidence for the prevalence of NSSI-related mental imagery in Borderline Personality Disorder (BPD) [15]. A substantial percentage (67%) of BPD patients reported NSSI related mental images, with nearly half (42%) being familiar with prospective rather than retrospective images, and 10% reporting both forms. Furthermore, one study identified increased rates of intrusive, prospective suicidal mental images in patients with BPD [32]. These images were comparable with the experience of suicidal imagination in patients with depression [33]. Finally, a recent study found that treating patients with BPD with imagery rescripting reduced BPD symptomatology, NSSI, intrusive prospective images, and depressive symptoms [14]. 

Based on previous scientific findings, it may be inferred that both NSSI and suicidality are the result of dysregulated emotions [34]. Moreover, a study on non-clinical students has indicated an interaction between mental imagery, NSSI, and affect [29]. In-depth knowledge about underlying mechanisms linking mental images and emotional dysregulation (i.e., negative emotional states) and psychopathology could be groundbreaking for the understanding of clinical disorders and the development of new therapeutic interventions.

Taking the previous studies together, there is preliminary evidence that negative affectivity may play a key role in linking mental images to psychopathology in patients with depression and BPD [7,8,14,15]. Moreover, even though there are comparatively few studies available on mental images in patients diagnosed with BPD, initial evidence points to the substantial clinical relevance of prospective mental images. Both self-harm and suicidal thoughts are core symptoms of the disorder [35], which have been linked to intrusive mental images [15,29,30,31]. 

Expanding the findings reported above, the present study aimed to investigate underlying mechanisms linking intrusive prospective imagery to psychopathology in patients diagnosed with BPD. Specifically, this is the first study to explore the possible mediating role of symptoms of depression as a proxy for negative affectivity. We also included a sample of patients diagnosed with depression as a comparison group, as mood instability, characterized by sadness, anxiety, frustration, as well as feelings of shame and guilt are particularly relevant to this population. Moreover, if considered a dimensional construct rather than a mere symptom, characteristics of depression can be found in both clinical disorders (i.e., BPD and depressive disorder [35]). However, as self-harm represents one of the major symptoms of BPD that does not typically occur in patients with depression or healthy subjects, we asked both groups to report on any other aversive, future-related, prospective mental image that is related to an upcoming distressing event (e.g., an upcoming exam). 

## 2. Material and Methods

### 2.1. Participants

The total sample consisted of 233 participants, out of whom 84 participants were diagnosed with major depressive disorders (MDD, F32, or F33) 66 participants were diagnosed with borderline personality disorder (BPD), and there were 83 healthy control participants (HC; for sample characteristics, see Table 1). Participants were included if they were at least 18 years old, fluent in oral and written German, and provided written informed consent.

Healthy control participants were excluded if they reported a prior history of mental health issues, a diagnosed psychiatric disorder (past or present), were admitted to an in- or outpatient psychiatric facility for treatment (past or present), or received psychotherapy (past or present). Participants diagnosed with depressive disorders were excluded if they met criteria for depression with psychotic symptoms, acute suicidality, schizophrenia spectrum disorder, neurological or organic psychosis, and BPD. BPD patients were excluded if they suffered from acute suicidality, a comorbid schizophrenia spectrum disorder, depression with psychotic symptoms, or neurological or organic psychosis (for comorbid disorders, see Table 2). Patients suffering from acute suicidality were excluded due to ethical reasons, as undergoing lengthy diagnostic interviews did not seem tenable under those circumstances, and patients would require immediate inpatient care. 

### 2.2. Instruments

German Impact of Future Events Scale—Short Version (IFES-S, [36]; English Original: [37]) consists of 20 items, measuring intrusive prospective imagery, hyperarousal, and avoidance. The German IFES-S [36] provides excellent internal consistency (Cronbach’s α = 0.93), comparable to its English counterpart (Cronbach´s α = 0.91; [37]). Within this questionnaire, healthy controls as well as patients suffering from depression were first asked to identify three future events that they had been imagining during the past seven days, and subsequently mark them as either positive or negative events. Thereafter, participants were asked to choose one negative event out of their created list and complete the IFES-S questionnaire whilst referring to this future negative event. Specifically, each participant responded to the 20 items regarding how much each of the statements applied to the negative prospective event that they had been imagining. Each item could be rated on a 5-point Likert scale (0 = not at all; 4 = extremely). The procedure was slightly altered for BPD patients, as they were not asked to identify three events, but rather to rate each of the 20 items referring to aversive, prospective NSSI-related mental images. This alteration was implemented as previous studies indicated that mental images are particularly relevant within the context of NSSI in BPD [15,27,28,30]. 

The Beck Depression Inventory—Second Edition (BDI-II; [38], German version) is a 21-item self-report measure, inquiring about symptoms of depression during the past 2 weeks, including the day of completion. Scores range from 0 to 63, with higher scores indicating severe symptoms of depression. The BDI-II provides a high internal consistency (Cronbach´s α = 0.84; [39]). The BDI-II was chosen as an instrument to evaluate negative affectivity, as the questionnaire assesses symptoms commonly associated with negative affect, such as sadness, pessimism, crying, or irritability. 

### 2.3. Procedure

Patients with depression or BPD were recruited through an outpatient psychiatric facility of the University Clinic of Ulm. Specifically, during the clinic´s intake assessment, they were asked if they were willing to partake in clinical studies. If they agreed, patients signed a consent form, in which they consented to have their contact information forwarded to the study clinician. Thereafter, the study clinician contacted the patient and conducted an initial screening in which inclusion criteria were examined, the purpose of the study was explained, and written informed consent was obtained. Afterwards, diagnoses were determined by clinical psychologists. All of these psychologists used the Structural Clinical Interview for DSM-IV Axis-I disorders (SCID-I; (41)) and Axis-II disorders (SCID-II; (41)) to evaluate the presence of clinical disorders. The SCID was conducted only to assess the clinical patients, who, thereafter, where included if they met criteria for either MDD or BPD as described above. The risks of partaking within the study were considered minimal, as patients were solely required to complete questionnaires as well as diagnostic interviews, which were routinely conducted within their inpatient care. Moreover, if patients provided written informed consent, test results were forwarded to their clinical therapist to enhance their inpatient treatment. 

Healthy controls were recruited at the University of Ulm, as well as within the general population. They were included based on self-reports stating that they did not suffer from a mental disorder (past or present) and had not received psychotherapeutic treatment (past or present). No standardized diagnostic assessment was conducted for the healthy control population. After accepting to participate in the study, all participants received an envelope containing several questionnaires, which they personally returned sealed to either the clinician of the study or their university professor after completion. As the questionnaires were distributed during a large-scale lecture (>200 students) held by an external professor, neither one of the included students were personally known by the lecturer.

### 2.4. Data Analysis

Data were analyzed using SPSS 29 [40]. All variables met the assumptions for conducting regression analysis. Means, percentages, and standard deviations were calculated to describe the control group, as well as the BPD and MDD groups on socio-demographic variables and comorbid disorders. Due to group differences within the socio-economic variables age, sex, and education, the variables were entered as covariates within the regression model. To test the hypothesis that negative affectivity may act as a mediator between intrusive prospective images and BPD symptomatology, regression analyses were performed using hierarchical linear modeling with the guidelines provided by Baron and Kenny (1986) and conducted using PROCESS Version 4.2 by Andrew F. Hayes for SPSS. A hierarchical regression analysis using intrusive prospective images as the outcome variable was performed by using groups as a predictor in the first step. In the second step, the significance of the relationship between the independent variable group and the mediator negative affectivity was established. In the third step, the significance of the relationship between the mediator negative affectivity and the dependent variable intrusive prospective images was evaluated. Lastly, we confirmed the meaningful reduction of the relationship between the independent variable group and the dependent variable intrusive prospective images in the presence of the mediator negative affectivity. Lastly, the Sobel test was performed to evaluate the significance of the pathway analysis.

## 3. Results

### 3.1. Baseline Characteristics

The three diagnostic groups differed in terms of age F(2216) = 53.36, *p* < 0.001; gender X2(2, N = 233) = 70.59, *p* < 0.001; educational level X2(2, N = 229) = 83.71, *p* < 0.001; negative affectivity (BDI) F(2) = 110.66, *p* < 0.001; and intrusive prospective images (IFES-S) F(2) = 31.35) *p* < 0.001 (Table 1).

### 3.2. Comorbid Disorders

Around 60% of patients within the depression group and 95% of patients within the BPD group fulfilled diagnostic criteria for additional mental disorders (see Table 2 for comorbid disorders). 

### 3.3. Mediation Analysis

**Figure 1:**
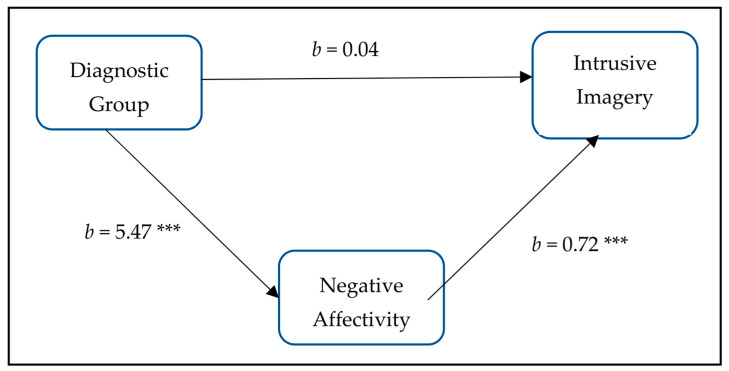
Mediation model showing the relation between diagnostic group, negative affectivity, and intrusive prospective imagery. Note: Diagnostic group = independent variable; Negative affectivity = mediator; Intrusive prospective imagery = dependent variable; *** = *p* < 0.001.

Multicollinearity was measured using variance inflation factors (VIF). Tests results indicated that multicollinearity was not a concern (Diagnostic Group, Tolerance = 0.73, VIF = 1.37; Negative Affectivity, Tolerance = 0.73, VIF = 1.37). Furthermore, autocorrelation in the model´s residuals was assessed using Durbin–Watson statistic, with values ranging from 0 to 4, and values around 2 indicating that there is no autocorrelation present and errors are independent. Henceforth, our data met the assumption of independent errors (Durbin–Watson value = 2.10). In the first step, the regression analysis revealed a main effect for the diagnostic group, displaying a positive relationship between the diagnostic group and intrusive prospective imagery (b = 5.68, t(201) = 3.56, *p* < 0.001). The second regression tested whether the variable diagnostic group predicted the mediator (i.e., negative affectivity). The variable diagnostic group was found to be positively associated with negative affectivity (b = 5.47, t(201) = 6.65, *p* < 0.001). For steps three and four, a regression analysis using prospective intrusive imagery as the outcome variable was performed using the variable diagnostic group as a predictor and negative affectivity as a moderator. The results indicated that there is a positive relationship between the mediator negative affectivity and the outcome variable intrusive prospective imagery even when controlled for the variable diagnostic group (b = 0.72, t(200) = 8.87, *p* < 0.001; see Figure 1). When negative affectivity (i.e., the mediator) was controlled for, the predictability for the variable diagnostic group was no longer significant for intrusive prospective imagery (b = 0.04, t(200) = 0.03, *p* = 0.98; see Figure 1). Negative affectivity improved the prediction of intrusive prospective imagery over and above the independent variable diagnostic group (R2 = 0.48, F(200) = 36.60, *p* < 0.001). The Sobel test was conducted to test the mediating criteria and to evaluate the significance of the indirect effect. Results revealed that the complete pathway from the diagnostic group (independent variable) to negative affectivity (mediator) to intrusive prospective imagery (dependent variable) was significant (z = 4.97; *p* < 0.001).

## 4. Discussion

The present study investigated intrusive mental imagery in BPD. Specifically, this is the first study to examine the mediating role of negative affectivity within the context of intrusive images in clinical patients. This study was based on previous findings demonstrating that affect has been associated with mental imagery and NSSI [29].

In terms of prospective intrusive imagery (as measured using IFES-S), the current findings demonstrated a link between the diagnostic group and intrusive prospective imagery. However, after controlling for negative affectivity (as measured using the BDI-II), the following results were obtained: the variable group no longer had a direct impact on intrusive prospective imagery. Rather, the link between the diagnostic group and intrusive prospective imagery was mediated by negative affectivity. Based on this relationship between negative affectivity and intrusive images, one might assume that negative affectivity is the key variable linking intrusive prospective imagery to psychopathology in clinical disorders such as BPD and depression. 

Within the context of previous theories on depression, our findings align with Beck´s theory [41]. Beck states that depressive disorders constitute of a negative view of the self, others, and the future [41]. To date, this theory has found support within various clinical studies investigating mental images. For example, several results have shown that depressed patients experience fewer positive but more negative prospective images when compared to mentally healthy control groups [16,42,43]. Moreover, [19] showed that depressed patients commonly experience distressing, intrusive mental images of either past or future events. 

On a similar note, mental images have been shown to elicit stronger emotions than a mere verbal processing of the same material [44]. At the same time, previous research has demonstrated that mental images can activate similar neuronal patterns as real-world perception [44]. Therefore, one could assume that imagining negative or intrusive scenarios might lead to an increase in depressive symptoms and distress in patients diagnosed with depression [7]. 

Connecting the above-mentioned theory on intrusive images and depression with our data on BPD, it is apparent that the current BPD sample exhibits an elevated prevalence (80%) of depressive disorders. This finding aligns with previous studies stating a similarly high comorbidity of depressive symptoms and BPD [45,46]. Furthermore, past research on BPD has found an association between suicidal images (also commonly experienced in depressed patients) and NSSI [32]. Therefore, depressive symptoms might be an indicator of negative affectivity and mood dysregulation across clinical disorders, linking prospective intrusive images to psychopathology. 

Based on this thought, prospective intrusive images could be regarded as a transdiagnostic phenomenon that occurs in a wide range of clinical conditions characterized by negative affectivity and mood dysregulation. In order to investigate this very preliminary hypothesis, prospective studies could assess negative affectivity or mood dysregulation within the context of prospective intrusive images in various clinical samples, such as bipolar disorders, anxiety disorders, or eating disorders. Furthermore, within clinical practice, it may be worthwhile to routinely assess prospective intrusive images in order to implement targeted interventions for intrusive images and their associated psychopathology. 

Our study presents some limitations, which should be discussed. First, participants were not matched in terms of age, gender, and educational level. This resulted in baseline differences between the investigated groups and could have impaired the presented results. However, these parameters have been added as covariates within the statistical model to account for the group differences. Second, healthy control participants did not undergo a diagnostic interview to objectively evaluate psychopathology. This might have impaired scientificity, as we cannot entirely guarantee that the information provided during self-report was accurate. Hence, to account for this limitation, future studies should conduct clinical interviews for all included populations. 

Furthermore, most patients suffering from depression or BPD experienced comorbid disorders such as anxiety or depressive disorders (BPD only). This is representative of the clinical populations as they appear in a naturalistic clinical setting. Nevertheless, due to these comorbidities, it might be difficult to draw clear-cut conclusions about the origin of intrusive prospective images. The reported intrusive images could have originated within the primary diagnosis (i.e., BPD or MDD) or within one of the comorbid disorders. Specifically, due to the high comorbidity of other Axis I disorders in patients with BPD, such as trauma related disorders or depression, it can be difficult to disentangle the relationship between BPD specific symptoms and other psychological constructs such as negative affectivity or intrusive prospective images. This could be because related symptoms (e.g., negative mood, intrusions) can occur in the context of any of the stated disorders. To account for these difficulties and distinguish the investigated groups, we excluded depressed patients who fulfilled diagnostic criteria for BPD. However, patients with BPD were allowed to display a comorbid diagnosis of depression, as most BPD patients also suffer from the latter. Moreover, patients with a comorbid trauma-related disorder were not excluded. Henceforth, it cannot be precluded that negative affectivity might have stemmed from some sort of trauma symptomatology. Therefore, future research on the comparison between BPD patients with- and without comorbid depressive and other Axis I disorders might be able to further distinguish underlying mechanisms by controlling for these disorders. Prospective studies could, hence, focus on including measures of various comorbid disorders, such as PTSD, when investigating intrusive images and BPD, as past research has shown its deleterious impact on the latter [32]. 

Lastly, while completing the questionnaire about prospective intrusive imagery (IFES-S), BPD patients were asked to imagine prospective acts of NSSI. However, the other two groups were asked to imagine a prospective negative event of any sorts. This procedure was implemented to investigate a most comparable scenario, as neither healthy controls nor depressed patients experienced NSSI. Nevertheless, future research could add a control group comprising participants who have practiced NSSI in the past or present but do not fulfill the diagnostic criteria for BPD for a more precise differentiation. 

## 5. Conclusions

This is the first study to investigate the mediating role of negative affectivity linking intrusive prospective images to psychopathology. Initially, the present results revealed an association between diagnostic group and intrusive prospective imagery. However, after controlling for negative affectivity, the results were significantly altered. The variable group no longer directly influenced intrusive prospective imagery. Instead, negative affectivity mediated the association. Therefore, it could be assumed that intrusive prospective images function as a transdiagnostic feature within clinical populations, where negative affectivity and mood instability are at the core of the disorder. 

The findings presented here are specifically relevant within the context of suicidality and NSSI. Negative affectivity and intrusive prospective images commonly co-occur during times of heightened distress. Therefore, the association between negative affectivity and intrusive prospective images might further increase the urge to execute associated behaviors such as NSSI. Even though this hypothesis must be treated with great caution, as the presented findings are preliminary, future studies could regularly address negative affectivity and intrusive prospective imagery in the context of NSSI and suicidality to deepen the knowledge about underlying mechanisms. In general, due to the possible effects of prospective intrusive imagery on actual behavior [13,15], future research is warranted with regard to a detailed investigation of prospective imagery in BPD, which may inform clinical practice and increase the usage of interventions targeting mental imagery in psychotherapy. 

## Figures and Tables

**Table 1:** Sample characteristics.

	Healthy Control Group (*n* = 83)	Depression Group (*n* = 84)	BPD Group (*n* = 62; 4 Missing)	
Sex				<0.001 ^1^
Female	74 (89.16%)	39 (46.43%)	66 (100%)	
Male	9 (10.84%)	45 (53.57%)	0 (0%)	
Education				<0.001 ^1^
No Degree	0 (0%)	0 (0%)	5 (7.9%)	
Secondary School Qualification	0 (0%)	17 (20.24%)	5 (6.3%)	
Extended Secondary School Qualification	3 (3.61%)	33 (39.29%)	22 (33.33%)	
High-School Diploma	23 (27.71%)	17 (20.24%)	10 (15.15%)	
University Degree	57 (68.67%)	17 (20.24%)	20 (30.30%)	
Age *	26.13 (5.31)	40.29 (13.66)	26.94 (6.95)	<0.001 ^2^
BDI *	5.25 (4.75)	22.15 (12.30)	30.46 (12.58)	<0.001 ^2^
IFES-S *	21.08 (12.78)	38.55 (16.98)	37.23 (16.83)	<0.001 ^2^

Note: BPD: Borderline Personality Disorder; ^1^ = Chi-Square Test; ^2^ = One-Way ANOVA; BDI: Beck Depression Inventory; IFES-S: Impact of Future Events Scale Short Version; * = Results are presented as Mean and (Standard Deviation).

**Table 2:** Comorbid disorders.

	Depression Group (*n* = 84)	BPD Group (*n* = 64; 2 Missing)
With Comorbid Disorder	51 (60.71%)	61 (95.31%)
Without Comorbid Disorder	33 (39.29%)	3 (4.69%)
Type of Comorbid Disorder *		
Major Depressive Disorder	Primary Diagnosis	51 (79.69%)
Anxiety Disorders	17 (20.24%)	44 (68.75%)
Obsessive-Compulsive Disorders	0 (0%)	10 (15.63%)
Trauma- and Stressor-related Disorders	7 (8.33%)	16 (25%)
Somatic Symptom and related Disorders	5 (5.95%)	0 (0%)
Eating Disorders	4 (5.76%)	12 (18.75%)
Sexual Dysfunctions	1 (1.19%)	0 (0%)
Substance-Related and Addictive Disorders	3 (3.57%)	14 (21.88%)
Personality Disorders	5 (5.95%)	N/A
Personality Disorder Traits	15 (17.86%)	N/A

Note: BPD = Borderline Personality Disorder; * Multiple entries were possible; N/A = not applicable.

## Data Availability

Data will be available from the corresponding author upon reasonable request.
